# Insomnia and Neuroticism in Pakistani Medical Students: A Cross-Sectional Study

**DOI:** 10.3390/healthcare13212778

**Published:** 2025-10-31

**Authors:** Sadia Qazi, Abdal Ahmad, Muhammad Awais Khan, Yameen Ahmed Qureshi, Muhammad Qasim, Hamza Farooq, Sara Shuaib, Laiba Irshad, Sanam Tajwali, Hamza Ali, Noman Ullah Wazir

**Affiliations:** 1 Department of Anatomy, College of Medicine, Alfaisal University, Riyadh 11533, Saudi Arabia; 2 MBBS Program, Peshawar Medical College, Riphah International University, Peshawar Campus, Peshawar 25160, Pakistansanamtajwali@gmail.com (S.T.); 3 Bannu Medical College, Bannu 28100, Pakistan; 4 Department of Anatomy, Peshawar Medical College, Riphah International University, Peshawar Campus, Peshawar 25160, Pakistan

**Keywords:** sleep disorders, insomnia severity, medical education, neuroticism, environmental moderation, student health

## Abstract

**Background**: Sleep disorders, particularly insomnia, represent a significant health concern in medical education. Neuroticism, characterized by emotional instability and stress reactivity, shows cross-sectional associations with sleep disturbances in healthcare trainees. Limited research examines these relationships among South Asian medical students. This cross-sectional study investigated insomnia symptom prevalence, personality correlates, and environmental factors among Pakistani medical students. **Methods**: We conducted a cross-sectional study among 364 undergraduate medical and dental students in Peshawar, Pakistan (June–November 2024). Data collection occurred during examination months. Data collection employed validated instruments: the Insomnia Severity Index (ISI) and the NEO Five-Factor Inventory neuroticism subscale (NEO-FFI-12). Statistical analyses included Pearson correlations, chi-square tests, and multivariate regression with interaction terms. **Results**: Among 364 participants (mean age 21.3 ± 2.3 years, 52.2% female), 47.0% reported severe insomnia symptoms (ISI 22–28), with 89.0% experiencing at least subthreshold symptoms (ISI ≥ 8) during the 2-week assessment period. These prevalence rates reflect symptom severity over a 2-week period during examination months and do not represent clinical diagnoses of chronic insomnia disorder, which requires ≥3 months of symptoms with clinical confirmation. High neuroticism (NEO-FFI ≥ 37) characterized 59.8% of students. Multivariate regression revealed a robust cross-sectional association between neuroticism and insomnia symptom severity (β = 0.239, 95% CI [0.173, 0.305], standardized β = 0.342, *p* < 0.001) and may reflect measurement during peak examination stress rather than stable trait-outcome relationships. Hostel residents showed non-significantly higher clinical insomnia prevalence than day scholars (75.9% vs. 67.5%, *p* = 0.081). Clinical-year students demonstrated significantly lower insomnia severity than pre-clinical students (β = −1.271, *p* < 0.001), a finding that contradicts assumptions about increasing stress through training progression. The neuroticism × living arrangement interaction was non-significant (*p* = 0.118); however, post hoc power analysis indicated the study was underpowered to detect small moderation effects, making this finding inconclusive. **Conclusions**: This study documents high insomnia symptom severity during a 2-week assessment period in Pakistani medical students, with a robust cross-sectional association with neuroticism. However, these findings must be interpreted within the constraints of the cross-sectional design, which cannot establish temporal precedence or causality between neuroticism and insomnia symptoms. These symptom prevalence rates likely reflect a combination of chronic sleep disorders and transient examination-related stress. Living arrangements showed small, non-significant associations with insomnia. The observed association between neuroticism and insomnia may be partially mediated or confounded by unmeasured variables, including academic stress, psychiatric comorbidities, substance use, and other sleep disorders. Findings suggest potential benefits from interventions addressing cognitive-emotional factors, though comprehensive diagnostic assessment is needed to distinguish chronic insomnia disorder from transient, stress-related sleep difficulties. Longitudinal research with objective sleep measures, structured psychiatric assessment, and systematic confounder evaluation is essential to establish causal relationships and intervention efficacy in this population.

## 1. Introduction

### 1.1. Sleep Disorders in Medical Education

Insomnia represents a prevalent sleep–wake disorder characterized by persistent difficulties with sleep initiation, maintenance, or early morning awakening, resulting in clinically significant daytime impairment despite adequate sleep opportunity [1]. This condition affects approximately 10–15% of the general adult population and has profound implications for cognitive performance, emotional regulation, and overall quality of life. The clinical manifestations of insomnia extend beyond nighttime sleep difficulties to encompass daytime consequences, including excessive sleepiness, reduced energy levels, irritability, mood disturbances, and impaired concentration. Insomnia can manifest as an acute condition lasting days to weeks, often triggered by specific stressors, or progress to a chronic disorder persisting for months or years. Chronic insomnia-related cognitive and psychomotor impairments increase accident risk and may compromise learning capacity and academic performance [1].

### 1.2. Global Prevalence of Sleep Disturbances in Medical Students

Medical education environments present unique challenges that may predispose students to sleep disturbances and related disorders. The demanding academic workload, irregular schedules, clinical rotations with overnight duties, and high-stress examination periods characteristic of medical training create conditions that may disrupt normal sleep–wake cycles. International research demonstrates concerning prevalence rates of sleep disorders among medical student populations. Studies from Jordan have documented hypersomnia prevalence ranging from 0.6% to 23.1% across different medical disciplines [2]. In the United States, 40–60% of college students experience sleep-related problems, with approximately one-third reporting inadequate sleep duration [3]. European studies have similarly identified significant sleep concerns, with research from Germany and Luxembourg revealing excessive daytime sleepiness in one-third of medical students [4].

Recent comprehensive meta-analyses of international medical student populations report poor sleep quality prevalence of 52.7% (95% CI: 45.3–60.1%) across 57 studies involving 25,735 students [5], with the most recent worldwide synthesis of 109 studies encompassing 59,427 students documenting 55.6% poor sleep quality and 33.3% excessive daytime sleepiness [6]. Within the Pakistani context, existing research has documented that 65.4% of medical students qualify as poor sleepers, with 49.4% experiencing excessive daytime sleepiness [7], indicating that sleep disturbances represent a substantial health concern in South Asian medical education settings.

### 1.3. Neuroticism and Its Association with Sleep Disturbances

Neuroticism, one of the fundamental dimensions of personality within the Five-Factor Model, represents a psychological factor that has been associated with sleep quality in multiple populations. This personality trait encompasses emotional instability, heightened stress reactivity, anxiety proneness, and increased vulnerability to negative emotional states. Individuals with elevated neuroticism demonstrate greater physiological arousal in response to stressors and exhibit persistent negative cognitive patterns, including worry, rumination, and catastrophic thinking, psychological processes that may interfere with sleep onset and maintenance mechanisms.

Research examining medical student populations across diverse cultural contexts has documented associations between neuroticism and psychological distress. Relationships between neuroticism, emotional regulation difficulties, and academic stress, with neuroticism demonstrating indirect effects on burnout through psychological distress pathways, have been documented [8]. The relationship between neuroticism and sleep disturbances appears bidirectional and complex, with emotional instability potentially contributing to sleep difficulties, while chronic sleep deprivation may exacerbate emotional dysregulation and stress reactivity.

### 1.4. The Intersection of Sleep, Personality, and Academic Performance

The intersection of personality traits, sleep disorders, and academic performance creates a complex web of associations with significant implications for medical student welfare and educational outcomes. Research has documented associations between neuroticism, emotional regulation difficulties, and academic stress in medical student populations, with neuroticism demonstrating significant indirect effects on burnout through psychological distress pathways [8]. Studies examining factors associated with depression in medical students have identified insomnia and neuroticism as significant correlates of depressive symptoms (*p* < 0.001), highlighting the interconnected nature of sleep, personality, and mental health in this population [9,10]. International research from China has demonstrated associations between personality factors and academic achievements in medical students, suggesting that personality traits may relate to learning outcomes [11]. Research examining personality-sleep relationships across populations has identified consistent associations between neuroticism and sleep disturbances [12]. These findings underscore the need for a comprehensive understanding of how personality factors and sleep disorders relate to academic success in medical education, though causal pathways remain to be established through longitudinal research.

### 1.5. Environmental Factors and Living Arrangements in South Asian Medical Education

Living arrangements during medical training may represent an additional environmental factor associated with sleep quality and stress levels. In Pakistan and South Asia broadly, medical students typically reside either in institutional hostels (dormitories) or commute daily from family homes, with these different living situations potentially creating distinct challenges for sleep hygiene and stress management.

Hostel environments in Pakistani medical institutions present unique characteristics shaped by cultural context. Gender-segregated dormitories typically house 2–4 students per room with shared facilities. These environments may expose students to increased ambient noise levels, irregular peer activities, social pressures, reduced family support, and limited control over environmental factors such as lighting and temperature. The communal nature of hostel living, while providing peer support opportunities, may also compromise privacy and personal sleep schedules. Additionally, hostel residents often experience separation from family support systems for the first time, potentially increasing psychological stress during the demanding medical training period.

Conversely, day scholars who commute from family homes face different challenges, including potentially lengthy travel times to campus (often 30–90 min each way in urban Pakistani settings), integration of family responsibilities with academic demands, and limited access to campus resources during evening hours. However, they may benefit from established family routines, familiar sleep environments, parental emotional support, and home-cooked meals.

Cultural factors specific to Pakistan further shape these living arrangements. The concept of pardah (gender segregation) influences hostel organization and social interactions. Family involvement in student decisions remains high, with varying preferences regarding residential arrangements. The month of Ramadan significantly disrupts sleep–wake cycles through pre-dawn meals (suhoor) and late-night prayers (taraweeh), with potential effects on circadian rhythms that may persist beyond the fasting period.

Limited research has systematically examined how these residential arrangements relate to personality traits and sleep outcomes in South Asian medical education contexts, representing an important gap in the literature.

### 1.6. Study Rationale and Knowledge Gaps

The present study addresses critical knowledge gaps in understanding the relationships between insomnia symptoms, personality factors, and environmental circumstances among medical and dental students in Pakistan. Given the documented high prevalence of sleep disturbances and the demanding nature of medical training, there is a need to identify factors associated with poor sleep to inform targeted interventions supporting student well-being.

This cross-sectional investigation examines associations between neuroticism, living arrangements, and insomnia symptoms. However, the cross-sectional design limits causal inference—we cannot determine whether neuroticism predisposes to insomnia, whether poor sleep increases emotional instability, or whether bidirectional relationships exist. Similarly, associations with living arrangements may reflect selection effects (students with certain characteristics choosing particular housing) rather than environmental causation. Longitudinal research is needed to establish temporal relationships and directional effects between personality traits, environmental factors, and sleep outcomes.

### 1.7. Study Objectives

This cross-sectional investigation seeks to (1) determine the prevalence of insomnia symptom severity levels and neuroticism among medical and dental students in Peshawar, Pakistan; (2) examine correlations between insomnia symptoms and neuroticism; (3) compare insomnia symptoms and neuroticism levels between hostel residents and day scholars; and (4) explore whether living arrangement moderates the neuroticism-insomnia relationship. These findings may inform hypotheses for future prospective research and preliminary considerations for intervention development, while acknowledging that cross-sectional associations cannot establish causation or predict intervention efficacy.

## 2. Methodology

### 2.1. Study Design and Setting

We conducted a comparative cross-sectional study over a six-month period (10 June 2024 to 5 November 2024). Data collection occurred at a single time point without follow-up; no interventions were administered. The study was conducted across medical and dental colleges in Peshawar, Pakistan. These institutions offer comprehensive undergraduate medical and dental training programs and include both government and private colleges.

### 2.2. Study Population and Sampling

#### 2.2.1. Target Population

The study population comprised undergraduate medical and dental students in Peshawar. All students from second year through final year who had successfully passed their professional examinations were eligible to participate.

#### 2.2.2. Sample Size Calculation

Based on a total population of 6650 eligible students, a sample size of 364 students was determined using the Raosoft sample size calculator with a 95% confidence interval and a 5% margin of error. This calculation ensures sufficient statistical power for the planned analyses.

#### 2.2.3. Sampling Method and Selection Criteria

A non-probability convenience sampling method was employed to recruit participants. Specifically, data collectors approached students during scheduled classroom periods within colleges of medicine and dentistry. All students present during scheduled data collection sessions were invited to participate. The number and timing of classroom sessions, departments contacted, and academic years represented during recruitment were determined based on investigator availability and institutional access. Students were excluded if they were not present in class during data collection periods or if they declined to provide consent to participate. This approach may systematically underrepresent students with higher absenteeism, introducing potential selection bias.

### 2.3. Data Collection Instruments

#### 2.3.1. Study Questionnaire

Data were collected using a structured, self-administered questionnaire comprising three main components: demographic information, the Insomnia Severity Index (ISI), and the neuroticism subscale of the NEO Five-Factor Inventory (NEO-FFI) in a paper format. The questionnaire was in English as it is the medium of instruction at the institutions.

#### 2.3.2. Insomnia Severity Index (ISI)

The ISI has seven items rated 0–4: total score 0–28. Interpretation: 0–7 no clinically significant insomnia, 8–14 subthreshold, 15–21 clinical (moderate), 22–28 clinical (severe) [13,14].

#### 2.3.3. Neuroticism (NEO-FFI) Assessment

Twelve items from the NEO-FFI neuroticism subscale were utilized to measure neuroticism levels. Responses were scored on a five-point Likert scale, with negatively worded items reverse-scored. Total score 12–60; higher scores indicate greater neuroticism [15]. Neuroticism categories were defined as follows: low neuroticism (<25), moderate neuroticism (25–36), and high neuroticism (≥37). These cut-offs were derived from published NEO-FFI normative data for comparable populations [15] and represent approximately 1 standard deviation above and below the mean, consistent with clinical interpretation practices for Five-Factor Model personality assessment.

#### 2.3.4. Academic Performance Evaluation

Academic performance was assessed using percentage-based categories: 60–85% and 85–100%, representing cumulative semester exam scores and coursework grades. These broad categories were used as a secondary exploratory variable; however, this crude measurement approach limits sensitivity for detecting correlations with insomnia or neuroticism compared to continuous GPA or standardized exam scores.

### 2.4. Pilot Testing and Reliability Assessment

Before main data collection, 10% of the calculated sample completed a pilot to assess reliability. Cronbach’s alpha was 0.78 for the ISI and 0.79 for the neuroticism subscale, exceeding the 0.70 threshold and indicating good internal consistency in this sample.

### 2.5. Ethical Considerations

The study protocol received approval from the Prime Foundation Institutional Review Board (Prime/ERC/2025-CRP011) on 15 June 2024. All procedures were conducted in accordance with the Declaration of Helsinki. Informed consent was obtained from each participant after explaining the study objectives and methodology. Participant anonymity was guaranteed, with no personal identifying information collected. Data confidentiality was maintained through password-protected electronic files accessible only to the lead investigator, and completed surveys were stored securely. Only aggregate statistics were presented to ensure individual participant responses could not be identified.

### 2.6. Statistical Analysis

Analyses used SPSS 25. Continuous variables are presented as mean ± SD; categorical variables as n (%). Group differences by residence were tested with χ^2^. Pearson correlations summarized associations between continuous measures. Multiple linear regression with the Insomnia Severity Index (ISI) as the primary outcome estimated associations between demographic and personality variables (neuroticism, age, gender, clinical year, living arrangement) and insomnia symptom severity, adjusting for demographics, with HC3 robust standard errors to address potential heteroscedasticity. A neuroticism × residence interaction term was tested to explore moderation effects. Model diagnostics included assessment of residual normality (Omnibus test), autocorrelation (Durbin–Watson statistic), and multicollinearity (variance inflation factors [VIF]). Statistical significance was set at two-sided α = 0.05.

## 3. Results

### 3.1. Participant Characteristics

A total of 364 students participated, achieving a 90% response rate among students present during scheduled data collection sessions. This response rate reflects only students accessible through classroom-based recruitment and does not account for students with chronic absenteeism, who may have experienced more severe sleep disturbances. Demographic characteristics are presented in Table 1. The mean age was 21.33 ± 2.26 years, with a female majority (52.2%). Most participants were enrolled in clinical training years (76.9%), attended private institutions (68.1%), and resided in hostels (53.6%). Academic performance was concentrated in the 60–85% range (76.4%), though this broad categorization represents a crude measurement of academic achievement.

### 3.2. Descriptive Statistics for Primary Variables

Table 2 presents descriptive statistics for continuous primary variables.

### 3.3. Insomnia Symptom Prevalence

**Figure 1:**
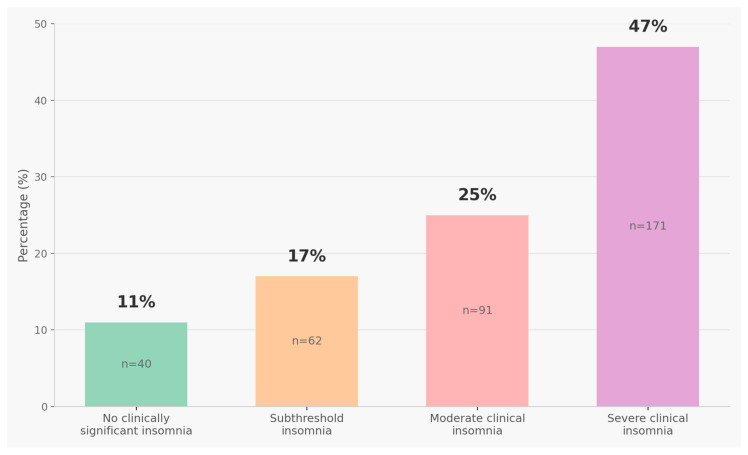
Distribution of Insomnia Symptom Severity Using Validated ISI Categories (n = 364). Bar chart showing distribution of insomnia symptom severity categories using validated Insomnia Severity Index (ISI) scoring. Values represent percentages of total participants, with sample sizes indicated for each category. Categories follow standard ISI interpretation: no clinically significant insomnia (0–7), subthreshold insomnia (8–14), moderate clinical insomnia (15–21), and severe clinical insomnia (22–28).

Analysis of insomnia symptom severity using validated ISI categories revealed high prevalence of sleep disturbances (Figure 1). Severe clinical insomnia (ISI 22–28) was present in 47.0% of students (n = 171), representing the largest proportion. Moderate clinical insomnia (ISI 15–21) affected 25.0% (n = 91), while subthreshold insomnia symptoms (ISI 8–14) were present in 17.0% (n = 62). Only 11.0% of students (n = 40) scored in the ‘no clinically significant insomnia’ range (ISI 0–7).

Overall, 89.0% of students (n = 324) experienced at least subthreshold insomnia symptoms (ISI ≥ 8), and 72.0% (n = 262) met criteria for clinical insomnia (ISI ≥ 15). These prevalence rates must be interpreted cautiously: they reflect insomnia symptom severity over a 2-week assessment period during examination months (June–November 2024), not clinical diagnoses of chronic insomnia disorder. Chronic insomnia disorder requires symptoms persisting for ≥3 months with clinical interview confirmation per DSM-5/ICSD-3 criteria [1]. The 47% severe insomnia prevalence likely represents an upper-bound estimate, conflating chronic insomnia with acute examination-related sleep disruption. Many students scoring in clinical ranges (ISI ≥ 15) may experience transient, stress-related sleep difficulties that resolve after examination periods rather than persistent insomnia disorder requiring intensive treatment (Figure 1).

### 3.4. Neuroticism Level Distribution

**Figure 2:**
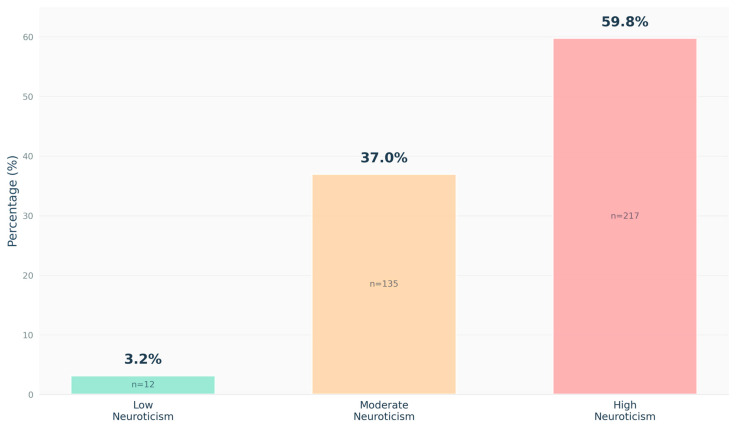
Distribution of Neuroticism Levels (n = 364). Bar chart showing distribution of neuroticism categories measured by NEO-FFI neuroticism subscale (score range 12–60). Categories defined as: low neuroticism (12–24), moderate neuroticism (25–36), high neuroticism (≥37). Values represent percentages of total participants with sample sizes indicated.

Assessment of neuroticism revealed elevated scores across the study population. High neuroticism (scores ≥ 37, representing approximately > 1 standard deviation above reported normative means in similar populations) characterized 59.8% of students (n = 217). Moderate neuroticism (scores 25–36) was observed in 37.0% (n = 135), while low neuroticism (scores < 25) was present in only 3.2% (n = 12). This distribution indicates that many medical students in this sample exhibited elevated trait neuroticism compared to population norms (Figure 2).

### 3.5. Comparative Analysis by Living Arrangement

#### 3.5.1. Insomnia Prevalence by Residence Type

Chi-square analysis examined differences in insomnia symptom prevalence between living arrangements (Table 3). Using ISI ≥ 8 as the threshold for any clinically relevant symptoms, hostel residents demonstrated small, non-significant prevalence compared to day scholars: 75.4% (n = 147) versus 69.2% (n = 117), χ^2^(1) = 1.579, *p* = 0.209. When examining clinical insomnia specifically (ISI ≥ 15), hostel residents showed 75.9% prevalence versus 67.5% among day scholars, χ^2^(1) = 3.047, *p* = 0.081. These differences represent small effect sizes (Cohen’s h = 0.14 and h = 0.19, respectively) and did not reach statistical significance at α = 0.05, suggesting that living arrangement has limited independent association with insomnia symptom severity in this sample.

#### 3.5.2. Neuroticism Levels by Residence Type

Neuroticism levels showed no significant difference between living arrangements: χ^2^(2) = 4.502, *p* = 0.069, Cramer’s V = 0.11. This marginally non-significant finding (*p* = 0.069) High neuroticism was observed in 56.9% of hostel residents and 62.7% of day scholars, demonstrating that personality trait expression is stable across living contexts and is not substantially influenced by residential arrangement. These results provide preliminary evidence that environmental factors (hostels vs. day scholar living) do not substantially moderate the neuroticism-insomnia relationship, consistent with the non-significant interaction term examined in the regression model (Section 3.7.2).

### 3.6. Correlation Analysis

**Figure 3:**
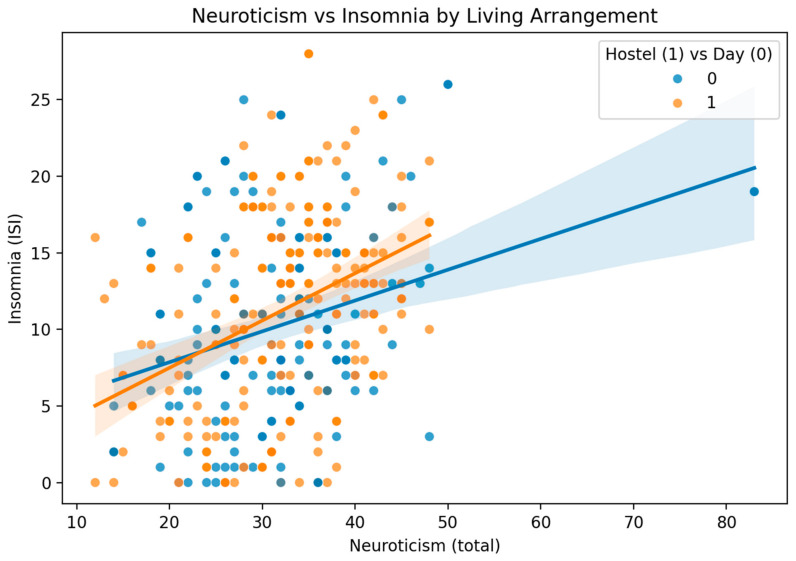
Association Between Neuroticism and Insomnia Symptom Severity (n = 364). Scatter plot displaying the positive correlation between neuroticism scores (NEO-FFI scale: 12–60) and insomnia symptom severity (ISI scores: 0–28) among undergraduate medical and dental students (n = 364). Blue circles represent day scholars (n = 169) and orange circles represent hostel residents (n = 195). Separate regression lines with 95% confidence intervals (shaded regions) are shown for each living arrangement group, demonstrating consistent positive associations in both populations. Overall correlation: r = 0.351, *p* < 0.001.

Pearson correlation analysis examined associations between study variables (Table 4). After Bonferroni correction (α = 0.017 for three comparisons), a significant moderate positive correlation was identified between insomnia severity and neuroticism (r = 0.351, *p* < 0.001), indicating shared variance of approximately 12.3% (r^2^ = 0.123)**.** To examine whether the neuroticism-insomnia correlation differed between living arrangements, Fisher’s z-test was conducted comparing the correlations for hostel residents (r = 0.342) and day scholars (r = 0.367). The correlations were not significantly different (z = 0.24, *p* = 0.808), indicating consistency of the personality-sleep relationship across residential contexts. This correlation remained stable across living arrangements (hostel residents: r = 0.342, *p* < 0.001; day scholars: r = 0.367, *p* < 0.001) (Figure 3).

### 3.7. Multivariate Regression Analysis

#### 3.7.1. Factors Associated with Insomnia Severity

Multiple linear regression examined factors associated with insomnia symptom severity while controlling for demographic variables (Table 5). The overall model was highly significant, F(5, 358) = 17.86, *p* < 0.001, explaining 16.7% of variance in insomnia severity (adjusted R^2^ = 0.155, ηp^2^ = 0.20).

Neuroticism was the strongest correlate of insomnia severity (unstandardized β = 0.239, standardized β = 0.342, 95% CI [0.173–0.305], *p* < 0.001). A one standard deviation increase in neuroticism (approximately 8.76 points) was associated with approximately 2.1 ISI points higher (95% CI [1.5, 2.7]), representing a small effect (Cohen’s d = 0.32).

Academic year was significantly associated with insomnia severity (β = −1.271, 95% CI [−1.826, −0.717], *p* < 0.001, d = 0.19), with clinical-year students reporting significantly lower symptom severity than pre-clinical students. This finding is noteworthy as it contradicts assumptions about increasing stress through medical training progression and may reflect selection effects (attrition of students with severe insomnia before reaching clinical years), adaptation to clinical responsibilities, or temporal variation in assessment timing relative to examination schedules.

Gender and living arrangement were not significantly associated with insomnia severity in the multivariate model (*p* = 0.473 and *p* = 0.073, respectively). Age showed a marginal positive association that did not reach statistical significance (β = 0.217, 95% CI [−0.006, 0.441], *p* = 0.056).

#### 3.7.2. Exploratory Interaction Effects Analysis

An exploratory interaction model examined whether living arrangement moderated the neuroticism-insomnia association (Table 6). The model including the neuroticism × living arrangement interaction term explained 17.1% of variance (adjusted R^2^ = 0.158), a modest 0.3% improvement over the main effects model.

The main effect of neuroticism remained significant (β = 0.190, 95% CI [0.102, 0.279], *p* < 0.001). The interaction term did not reach statistical significance (β = 0.100, 95% CI [−0.025, 0.225], *p* = 0.118), indicating insufficient evidence that living arrangement moderates the neuroticism-insomnia relationship in this sample.

Post hoc power analysis was conducted to contextualize this non-significant moderation finding. The interaction term explained an additional 0.4% of variance (ΔR^2^ = 0.004), corresponding to a very small effect size (Cohen’s f^2^ = 0.0048). To detect a small interaction effect (f^2^ = 0.02) at 80% power with α = 0.05 and six predictors, a sample size of approximately n = 400 would be required. The present study (n = 364) was therefore underpowered by approximately 36 participants (9%) for detecting small moderation effects. The observed power to detect f^2^ = 0.02 with n = 364 was approximately 58–65%. Consequently, the non-significant interaction (*p* = 0.118) may reflect insufficient statistical power rather than genuine absence of effect modification by living arrangement. Larger studies or multisite research would be necessary to definitively establish whether living arrangement moderates the neuroticism-insomnia relationship.

#### 3.7.3. Model Diagnostics

Diagnostic testing indicated the regression models satisfied key statistical assumptions. Durbin-Watson statistics (1.891–1.902) indicated no significant autocorrelation in residuals, confirming independence of observations. The Omnibus test suggested minor deviation from perfect normality (χ^2^ = 9.677, *p* = 0.008), a common finding in psychological research with non-normal distributions and appropriately addressed through the use of HC3 robust standard errors, which do not assume normality and provide valid inference even with minor deviations from this assumption. Variance inflation factors ranged from 1.12 to 2.18 (all < 2.5), indicating no problematic multicollinearity and confirming that predictor variables are sufficiently independent. Visual inspection of residual plots confirmed appropriateness of linear modeling assumptions. Collectively, these diagnostics support the validity of the regression coefficients and model-based inferences presented in Section 3.7.1 and Section 3.7.2.

## 4. Discussion

### 4.1. Principal Findings and Prevalence Context

This cross-sectional study of 364 Pakistani medical and dental students reveals three principal findings: (1) exceptionally high prevalence of severe insomnia symptoms (47.0%), with 89% experiencing at least subthreshold symptoms; (2) a robust cross-sectional association between neuroticism and insomnia severity, with moderate effect size (r = 0.351, β = 0.342); and (3) modest environmental associations, with hostel residents showing non-significantly higher clinical insomnia prevalence than day scholars (75.9% vs. 67.5%, *p* = 0.081).

The 47% severe insomnia symptom prevalence (ISI ≥22) substantially exceeds general adult population rates of clinical insomnia (10–15%) [1]. However, direct comparisons require caution: the ISI assesses insomnia symptoms specifically, whereas most international research uses the Pittsburgh Sleep Quality Index (PSQI) to measure broader sleep quality constructs. These represent distinct assessment approaches. Recent comprehensive meta-analyses report poor sleep quality prevalence of 52.7% (95% CI: 45.3–60.1%) in 25,735 medical students across 57 studies [5], with the most recent worldwide synthesis of 109 studies involving 59,427 students reporting 55.6% (95% CI: 51.45–59.74%) poor sleep quality and 33.3% (95% CI: 26.52–40.91%) excessive daytime sleepiness [6]. These PSQI-based estimates represent different constructs than ISI-measured insomnia severity but suggest widespread sleep disturbances among medical trainees globally.

The high prevalence observed in our Pakistani sample converges with findings from other South Asian and Middle Eastern medical education contexts, including South India (62% sleep disturbances) [16] and Saudi Arabia (>50% concurrent insomnia-anxiety) [17]. This pattern suggests systematic factors specific to medical training in these regions [18], potentially including higher academic pressure [19], limited mental health support infrastructure, cultural attitudes toward help-seeking [20], and environmental stressors (e.g., hostel living, diet) [21] contribute to sleep problems and psychological distress. Previous Pakistani research also supports this, reporting 65.4% poor sleepers [7], with similar high academic stress and anxiety levels documented in Saudi and Pakistani cohorts [22,23,24] in educational settings. However, critical interpretive caution is warranted. These prevalence rates must be interpreted as reflecting insomnia-level symptom severity over a 2-week assessment period during examination months (June–November 2024), not clinical insomnia diagnoses requiring ≥ 3-month symptom duration and clinical interview confirmation per DSM-5/ICSD-3 criteria [1]. The 47% severe insomnia prevalence likely represents an upper-bound estimate during peak examination stress periods, conflating chronic insomnia disorder with acute examination-related sleep disruption. Many students scoring in clinical ranges (ISI ≥ 15) may have stress-related, situational sleep difficulties that resolve with academic schedule changes, rather than persistent insomnia requiring intensive behavioral or pharmacological treatment. Distinguishing between acute stress-response insomnia and chronic insomnia disorder has important implications for intervention planning and resource allocation.

Our findings should be interpreted as indicating that during examination-heavy academic periods, nearly half of medical students experience severe insomnia-level symptoms, rather than implying that 47% have chronic insomnia disorder requiring clinical intervention. Future research employing longitudinal designs with repeated assessments across full academic calendars, diagnostic interviews, and objective sleep measures (polysomnography, actigraphy) would provide more definitive prevalence estimates and clarify the proportion of students with persistent versus transient sleep difficulties.

### 4.2. Neuroticism-Insomnia Association: Mechanisms and Interpretation

The neuroticism-insomnia correlation (r = 0.351, explaining 12.3% of shared variance) aligns with established personality-sleep research. The standardized effect (β = 0.342) indicates that a one standard deviation increase in neuroticism is associated with approximately 2.1 ISI points, a small effect (Cohen’s d = 0.32) that could shift some individuals from subthreshold to clinical categories, though most would remain within their original severity category.

Critically, the cross-sectional design precludes causal inference. While personality traits show relative stability, chronic sleep deprivation may transiently increase emotional lability and stress reactivity, potentially inflating neuroticism scores [19]. Additionally, unmeasured confounders (psychiatric comorbidity, substance use, other sleep disorders) may substantially mediate or confound the observed association rather than representing a direct personality effect [25]. The bidirectional and potentially reciprocal nature of this relationship means we cannot determine whether (1) neuroticism predisposes to insomnia development, (2) poor sleep exacerbates emotional instability, (3) bidirectional and reinforcing relationships exist, or (4) third variables (e.g., academic stress, life circumstances) drive both [26].

Several potentially mechanistic pathways may explain this observed association. Existing evidence linking personality factors, coping, and academic performance supports the need for longitudinal designs [27,28,29]. Cognitively, individuals high in neuroticism demonstrate greater pre-sleep cognitive arousal through rumination, worry, and catastrophic thinking about sleep difficulties themselves, potentially creating cycles of sleep-related anxiety and insomnia symptoms [30]. For example, Slavish et al. (2018) demonstrated in longitudinal research that rumination mediates the neuroticism-sleep relationship, with daily fluctuations in rumination predicting same-night sleep quality [30]. Neurobiologically, elevated neuroticism correlates with chronic HPA axis hyperactivity, elevated evening cortisol, and sustained sympathetic nervous system activation, physiological states that may interfere with sleep initiation [25]. Finally, recent meta-analyses demonstrate consistent associations between neuroticism and poor sleep quality across populations, with pooled effect sizes in the r = 0.25–0.35 range [31]. However, these mechanistic interpretations are speculative in the current cross-sectional design and await confirmation through prospective research.

### 4.3. Environmental Factors: Living Arrangements and Cultural Context

Hostel residents demonstrated non-significantly higher clinical insomnia prevalence compared to day scholars (75.9% vs. 67.5%, *p* = 0.081, h = 0.19). This small effect suggests living arrangements account for limited variance in sleep outcomes compared to individual factors like personality. These findings provide preliminary evidence that environmental factors (hostel vs. day scholar living) contribute minimally to insomnia severity independent of personality factors.

The exploratory interaction analysis did not reach statistical significance (*p* = 0.118), providing insufficient evidence that living arrangement moderates the neuroticism-insomnia relationship. However, the study was underpowered to detect small interaction effects. Larger studies are needed to definitively test this hypothesis.

Hostel environments in Pakistani medical institutions present environmental stressors that may contribute to sleep disruption, including gender-segregated dormitories housing multiple students per room, limited environmental control over noise and lighting, separation from family support systems, and integration into peer networks with potentially conflicting sleep schedules. Cultural factors, including pardah (gender segregation), create distinct social dynamics for male versus female students. However, substantial selection effects complicate causal interpretation: students choosing hostel residence may differ systematically from those living at home in unmeasured characteristics (resilience, coping strategies, symptom severity at baseline) that affect both personality expression and sleep outcomes [27,28,29,32]. Cross-sectional comparisons cannot disentangle environmental influences from selection processes.

The timing of data collection (June–November 2024, following Ramadan in March-April) warrants consideration. Although data collection occurred 2–5 months post-Ramadan, residual circadian rhythm effects from the altered sleep–wake schedules during Ramadan cannot be entirely excluded, particularly if students maintained modified sleep patterns into the data collection period.

### 4.4. Academic Training Progression: Alternative Explanations

Clinical-year students reported significantly lower insomnia symptom severity than pre-clinical students (β = −1.271, *p* < 0.001, d = 0.19). This finding challenges assumptions about increasing stress through medical training progression. Multiple plausible alternative explanations warrant consideration beyond “protective effects of clinical training”:

- Students experiencing severe insomnia and associated functional impairments may disproportionately withdraw from medical training before reaching clinical years, resulting in healthier clinical cohorts. Our cross-sectional design cannot address this possibility.
- Clinical students are older (confounded with academic year), and age showed marginal positive association with insomnia (*p* = 0.056), creating complex relationships.
- Successful progression may reflect development of effective stress management strategies that secondarily benefit sleep [27,28,29], rather than clinical training itself providing protection.
- If data collection occurred during particularly stressful pre-clinical examination periods but routine clinical rotation periods, temporal variation in stress exposure could explain differences.
- Only students who successfully pass professional examinations progress to clinical years, potentially selecting for resilience factors.

These competing mechanisms cannot be distinguished with cross-sectional data. Longitudinal designs following individual students from matriculation through graduation are needed to establish whether insomnia symptoms genuinely decrease over time or whether between-group differences reflect selection processes.

### 4.5. Clinical Implications for Sleep Medicine Practice

The 89% prevalence of at least subthreshold insomnia symptoms and 72% prevalence of clinical-level symptoms (ISI ≥ 15) indicate that Pakistani medical students represent a high-need population for sleep medicine services. However, several considerations temper intervention recommendations:

Diagnostic clarification needed: ISI scores indicate symptom severity but not clinical diagnosis. Many students scoring in clinical ranges may have transient, stress-related sleep difficulties rather than chronic insomnia disorder. Comprehensive assessment, including duration, sleep opportunity, and differential diagnosis, is essential before treatment.

Adapted interventions: Standard Cognitive Behavioral Therapy for Insomnia (CBT-I) may require modification for medical student contexts, including irregular schedules, clinical rotations, and shared living spaces. Adaptations might include a focus on circadian rhythm alignment for shift work, strategies for managing “on-call” sleep disruption, or brief, app-based interventions for scalable delivery. Digital CBT-I interventions offer scalable delivery methods for college student populations.

Integrated approaches: The moderate neuroticism-insomnia association suggests that interventions addressing cognitive-emotional factors (rumination reduction, stress inoculation, emotional regulation skills) may complement sleep-focused treatments. Mindfulness-based interventions show promise for high-neuroticism individuals [25].

Institutional collaboration: Multi-tier screening during routine health evaluations, streamlined referral pathways, and environmental modifications (hostel noise reduction policies, quiet hours, and sleep hygiene education) may provide population-level benefits.

### 4.6. Study Limitations

The fundamental limitation is the cross-sectional design, which precludes establishing temporal precedence or determining causal direction between neuroticism and insomnia. Specifically, we cannot determine whether neuroticism predisposes to insomnia, whether chronic insomnia exacerbates emotional reactivity, or whether both are driven by unmeasured stressors. All associations are correlational and compatible with multiple causal models.

Reliance on questionnaires introduces potential biases, including social desirability, recall inaccuracy, and response styles. Absence of objective sleep measures (polysomnography, actigraphy) prevents validation of subjective reports or assessment of sleep architecture. Perceived insomnia severity may not correspond to objectively measured sleep disruption.

Convenience sampling during classroom sessions underrepresents students with high absenteeism, who may have worse sleep. This selection bias likely underestimates true prevalence and insomnia severity. The 90% response rate among present students does not address systematic absence bias.

A single-city sample (Peshawar) limits generalizability to other Pakistani regions or international contexts with different cultural, socioeconomic, and educational characteristics.

The 2-week ISI timeframe may capture transient examination-driven sleep disruption rather than stable insomnia patterns. Since data collection occurred during examination periods (June–November 2024), the high prevalence rates (47% severe insomnia) may represent an upper-bound estimate reflecting acute stress responses rather than chronic insomnia disorder. Academic calendar timing of data collection creates temporal specificity.

The study had limited statistical power to detect small interaction effects, contributing to the non-significant moderation finding (*p* = 0.118).

Academic stress, life events, mental health disorders, medical conditions, substance use (caffeine, stimulants), and other sleep disorders were not systematically assessed. These variables independently associate with both insomnia and neuroticism. Consequently, the observed neuroticism-insomnia correlation may be substantially confounded or mediated by unmeasured psychiatric conditions, stress, or other sleep pathology rather than representing a direct personality effect. The magnitude of bias introduced by these unmeasured confounders is unknown.

### 4.7. Future Research Directions

Longitudinal studies following students from matriculation through graduation would establish temporal sequences, determine causal directionality, and test competing mechanistic models. Concurrent assessment of academic stress, psychiatric status, comorbid sleep disorders, substance use patterns, and life events would permit mediation and stratification analyses clarifying whether confounders explain the observed neuroticism-insomnia association.

Rigorous objective assessment incorporating polysomnography and actigraphy alongside clinical interviews and comprehensive psychiatric evaluation would validate ISI scores and clarify mechanistic pathways. Randomized controlled trials testing adapted CBT-I protocols, mindfulness-based interventions, and environmental modifications specifically for medical student populations. Such trials must occur post-intervention refinement informed by longitudinal confounder data.

Investigation of mechanisms linking neuroticism to insomnia (rumination, pre-sleep cognitive arousal, dysfunctional sleep beliefs, emotion regulation deficits, HPA axis reactivity) to identify intervention targets. Experience sampling and daily diary designs tracking within-person fluctuations in stress, emotion, and sleep would illuminate causal temporal sequences.

Comparative studies across regions with varying cultural attitudes toward sleep, educational practices, and support systems. Multi-site designs would establish generalizability and identify region-specific factors influencing the neuroticism-insomnia relationship.

## 5. Conclusions

This study documents exceptionally high prevalence of insomnia symptoms among Pakistani medical students (89% with at least subthreshold symptoms, 47% with severe symptoms), with a robust cross-sectional association between neuroticism and insomnia symptom severity (r = 0.351, β = 0.342). However, these findings must be interpreted within the constraints of the cross-sectional design, which cannot establish temporal precedence, causal direction, or whether the observed association is direct or mediated by unmeasured confounders, including psychiatric comorbidity, academic stress, substance use, or other sleep disorders. High prevalence rates reflect insomnia symptom severity measured over a 2-week period during potentially high-stress examination months and may reflect both transient stress-related sleep difficulties and chronic insomnia disorder, but the distinction cannot be determined without clinical diagnostic interviews and longer-term follow-up. Living arrangements showed small, non-significant associations with insomnia prevalence, and exploratory moderation analysis did not reach statistical significance (*p* = 0.118).

Preliminary findings indicate that medical students could potentially benefit from targeted sleep health interventions, particularly those addressing cognitive-emotional factors associated with neuroticism. However, whether neuroticism causally contributes to insomnia development, whether insomnia exacerbates neuroticism, or whether shared vulnerability to stress mediates both remains unknown from cross-sectional data. Comprehensive diagnostic assessment is needed to distinguish students with chronic insomnia disorder requiring intensive treatment from those with transient, stress-related sleep difficulties who may benefit from psychoeducation and sleep hygiene guidance. Longitudinal research employing objective sleep measures, structured psychiatric assessment, and comprehensive evaluation of potential confounders is essential to establish temporal relationships, determine causal pathways, and definitively evaluate intervention efficacy in this population.

## Figures and Tables

**Table 1:** Demographic Characteristics of Study Participants (n = 364).

Variable	Category	n (%)
Age	Mean ± SD	21.33 ± 2.26 years
Gender	Male	174 (47.8%)
	Female	190 (52.2%)
Academic Level	Pre-clinical (Years 2–3)	84 (23.1%)
	Clinical (Years 4–5)	280 (76.9%)
Institution Type	Public	116 (31.9%)
	Private	248 (68.1%)
Living Arrangement	Hostel resident	195 (53.6%)
	Day scholar	169 (46.4%)
Academic Performance	35–60%	35 (9.6%)
	60–85%	278 (76.4%)
	85–100%	51 (14.0%)

**Table 2:** Descriptive Statistics for Insomnia Severity and Neuroticism (n = 364).

Variable	Mean ± SD	Median	Range	Skewness	Kurtosis
ISI Total Score	17.82 ± 6.53	18.0	3–28	−0.21	−0.69
Neuroticism Score	32.45 ± 8.76	33.0	13–58	0.08	−0.42

ISI = Insomnia Severity Index; SD = standard deviation.

**Table 3:** Comparison of Insomnia Symptoms and Neuroticism by Living Arrangement.

Variable	Category	Hostel Residents n (%)	Day Scholars n (%)	χ^2^	*p*-Value	Effect Size
Any Insomnia Symptoms (ISI ≥ 8)	Present	147 (75.4%)	117 (69.2%)	1.579	0.209	h = 0.14
	Absent	48 (24.6%)	52 (30.8%)			
Clinical Insomnia (ISI ≥ 15)	Present	148 (75.9%)	114 (67.5%)	3.047	0.081	h = 0.19
	Absent	47 (24.1%)	55 (32.5%)			
Neuroticism Level	High	111 (56.9%)	106 (62.7%)	4.502	0.069	V = 0.11
	Moderate/Low	84 (43.1%)	63 (37.3%)			

h = Cohen’s h; V = Cramer’s V; None of the comparisons reached statistical significance at α = 0.05.

**Table 4:** Pearson Correlation Matrix for Study Variables (n = 364).

Variable	ISI Score	Neuroticism
ISI Score	1.000	-
Neuroticism	0.351 **	1.000

** *p* < 0.001 (survives Bonferroni correction at α = 0.017), ISI = Insomnia Severity Index.

**Table 5:** Multiple Regression: Factors Associated with Insomnia Severity.

Predictor	Unstandardized β (95% CI)	Standardized β	*p*-Value	Cohen’s d
Intercept	0.754 (−4.125, 5.633)	-	0.762	-
Neuroticism	0.239 (0.173, 0.305)	0.342	<0.001 *	0.32
Age	0.217 (−0.006, 0.441)	0.075	0.056	-
Female	0.470 (−0.813, 1.753)	0.036	0.473	-
Clinical Year	−1.271 (−1.826, −0.717)	−0.097	<0.001 *	0.19
Hostel Resident	1.179 (−0.109, 2.467)	0.090	0.073	-

Model Statistics: R^2^ = 0.167; Adjusted R^2^ = 0.155, F(5, 358) = 17.86, *p* < 0.001, ηp^2^ = 0.20; β = regression coefficient; * *p* < 0.05 for statistical significance; CI = confidence interval; d = Cohen’s d for significant effects. HC3 robust standard errors used.

**Table 6:** Exploratory Interaction Model: Neuroticism × Living Arrangement.

Predictor	β (95% CI)	*p*-Value
Neuroticism	0.190 (0.102, 0.279)	<0.001 *
Age	0.239 (0.027, 0.451)	0.027 *
Female	0.504 (−0.781, 1.789)	0.442
Clinical Year	−1.262 (−1.809, −0.714)	<0.001 *
Hostel Resident	−2.025 (−6.265, 2.214)	0.349
Neuroticism × Hostel	0.100 (−0.025, 0.225)	0.118

Model Statistics: R^2^ = 0.171; Adjusted R^2^ = 0.158, ΔR^2^ = 0.004 (compared to main effects model), F(6, 357) = 16.65, *p* < 0.001, * *p* < 0.05 for statistical significance.

## Data Availability

The original contributions presented in this study are included in the article/Supplementary Materials. Further inquiries can be directed to the corresponding author.

## Supplementary Materials

The following supporting information can be downloaded at: https://www.mdpi.com/article/10.3390/healthcare13212778/s1, Study questionnaire including Insomnia Severity Index (ISI) and NEO Five-Factor Inventory neuroticism subscale (NEO-FFI-12) is available as Supplementary File.
