# Supplementary material for: Insomnia and Neuroticism in Pakistani Medical Students: A Cross-Sectional Study
Source: Healthcare (Basel). 2025 Oct 31;13(21):2778. doi: 10.3390/healthcare13212778 (PMC12609372; doi:10.3390/healthcare13212778)
Supplement: Supplementary file 1 [file healthcare-13-02778-s001.zip › healthcare-3939434-supplementary.pdf]

Supplementary file: Insomnia and Neuroticism in Pakistani Medical Students: A Cross-Sectional Study

STUDY QUESTIONNAIRE
IRB Approval: Prime Foundation Institutional Review Board (Prime/ERC/2025-CRP011)
Estimated Completion Time: 15-20 minutes

INFORMED CONSENT

Study Purpose

This research investigates sleep patterns and personality traits among medical and dental students to better understand factors that may affect student wellbeing.

What Participation Involves

You will be asked to complete a questionnaire about your sleep patterns, personality characteristics, and basic demographic information. The questionnaire takes approximately 15-20 minutes to complete.

Risks and Benefits

There are no direct benefits to you from participation. Some questions address sleep difficulties and emotional experiences that may cause mild discomfort. If you experience distress while completing this questionnaire, you may stop at any time and seek support from the resources listed at the end of this form.

Confidentiality

Your responses are completely confidential and anonymous. No personal identifying information will be collected. Data will be stored securely with password protection and only aggregate statistics will be reported.

Voluntary Participation

Participation is entirely voluntary. You may decline to participate or withdraw at any time without any penalty or negative consequences for your academic standing.

Consent Statement

Do you consent to participate in this research study?

☐ Yes, I consent to participate. I have read the above information and understand that participation is voluntary. I agree to participate in this study.

☐ No, I do not consent to participate.

If you do not consent, please return this form blank. If you consent, please continue with the questionnaire below.

SECTION 1: DEMOGRAPHIC INFORMATION

1. Gender:

☐ Male

☐ Female

☐ Prefer not to answer

2. Age: _________ years

3. Academic Year:

☐ Second year (pre-clinical)

☐ Third year (pre-clinical)

☐ Fourth year (clinical)

☐ Fifth year (clinical)
4. Program:
☐ Medical (MBBS)
☐ Dental (BDS)
5. Institution Type:
☐ Public institution
☐ Private institution
6. Living Arrangement:
☐ Hostel resident (living in university dormitory)
☐ Day scholar (living at home with family)
7. Academic Performance (Most Recent Professional Examination):
☐ 85-100%
☐ 70-84%
☐ 60-69%
☐ 50-59%
☐ Below 50%
☐ Prefer not to answer

SECTION 2: INSOMNIA SEVERITY INDEX (ISI)

Instructions: For each question, please mark the number that best describes your experience during the past 2 weeks, including last night.

Sleep Difficulty Assessment

1.Difficulty falling asleep

Not at all	A Little	Somewhat	Much	Very Much
☐ 0	☐ 1	☐ 2	☐ 3	☐ 4

2. Difficulty staying asleep

Not at all	A Little	Somewhat	Much	Very Much
☐ 0	☐ 1	☐ 2	☐ 3	☐ 4

3. Problems waking up too early

Not at all	A Little	Somewhat	Much	Very Much
☐ 0	☐ 1	☐ 2	☐ 3	☐ 4

Sleep Impact Assessment

4. How satisfied/dissatisfied are you with your current sleep pattern?

Not at all	A Little	Somewhat	Much	Very Much
☐ 0	☐ 1	☐ 2	☐ 3	☐ 4

5. How noticeable to others do you think your sleep problem is in terms of impairing the quality of your life?

Not at all	A Little	Somewhat	Much	Very Much
☐ 0	☐ 1	☐ 2	☐ 3	☐ 4

6. How worried/distressed are you about your current sleep problem?

Not at all	A Little	Somewhat	Much	Very Much
☐ 0	☐ 1	☐ 2	☐ 3	☐ 4

7. To what extent do you consider your sleep problem to interfere with your daily functioning (e.g., daytime fatigue, mood, ability to function at work/daily chores, concentration, memory)?

Not at all	A Little	Somewhat	Much	Very Much
☐ 0	☐ 1	☐ 2	☐ 3	☐ 4

SECTION 3: PERSONALITY ASSESSMENT (NEO-FFI NEUROTICISM SUBSCALE)
Instructions: Please indicate how much you agree or disagree with each statement. Some statements are worded positively and some negatively—please read each one carefully.

#	Statement	Strongly Disagree	Disagree	Neutral	Agree	Strongly Agree
1	I often feel inferior to others.	☐ 1	☐ 2	☐ 3	☐ 4	☐ 5
2	When I'm under a great deal of stress, sometimes I feel like I'm going to pieces.	☐ 1	☐ 2	☐ 3	☐ 4	☐ 5
3	I often feel tense and jittery.	☐ 1	☐ 2	☐ 3	☐ 4	☐ 5
4	Sometimes I feel completely worthless.	☐ 1	☐ 2	☐ 3	☐ 4	☐ 5
5	I often get angry at the way people treat me.	☐ 1	☐ 2	☐ 3	☐ 4	☐ 5
6	Too often, when things go wrong, I get discouraged and feel like giving up.	☐ 1	☐ 2	☐ 3	☐ 4	☐ 5
7	I often feel helpless and want someone else to solve my problems.	☐ 1	☐ 2	☐ 3	☐ 4	☐ 5
8	At times I have been so ashamed I just want to hide.	☐ 1	☐ 2	☐ 3	☐ 4	☐ 5
9	I am not a worrier.	☐ 1	☐ 2	☐ 3	☐ 4	☐ 5
10	I rarely feel lonely or blue.	☐ 1	☐ 2	☐ 3	☐ 4	☐ 5
11	I rarely feel fearful or anxious.	☐ 1	☐ 2	☐ 3	☐ 4	☐ 5
12	I am seldom sad or depressed.	☐ 1	☐ 2	☐ 3	☐ 4	☐ 5

Thank you for your participation in this research study. Your responses will contribute to better understanding of student wellbeing and may inform future support services.
Please return this completed questionnaire to the research team member.

Note: This questionnaire is for research purposes only and should not be used for clinical diagnosis.

Instrument References

1. Bastien CH, Vallières A, Morin CM. Validation of the insomnia severity index as an outcome measure for insomnia research. Sleep Med. 2001;2(4):297-307.

2. Morin CM, Belleville G, Bélanger L, Ivers H. The Insomnia Severity Index: psychometric indicators to detect insomnia cases and evaluate treatment response. Sleep. 2011;34(5):601-8.
3. Costa PT, McCrae RR. Revised NEO Personality Inventory (NEO-PI-R) and NEO Five-Factor Inventory (NEO-FFI) professional manual. Odessa, FL: Psychological Assessment Resources; 1992.
